# Reverse Auction: A Potential Strategy for Reduction of Pharmacological Therapy Cost

**DOI:** 10.5935/abc.20150076

**Published:** 2015-09

**Authors:** Sara Michelly Gonçalves Brandão, Victor Sarli Issa, Silvia Moreira Ayub-Ferreira, Samantha Storer, Bianca Gigliotti Gonçalves, Valter Garcia Santos, Nelson Carvas Junior, Guilherme Veiga Guimarães, Edimar Alcides Bocchi

**Affiliations:** Instituto do Coração do Hospital das Clínicas da Faculdade de Medicina da Universidade de São Paulo, SP - Brazil

**Keywords:** Heart Failure, Pharmaceutical Preparations / economics, Competitive Bidding / economics, Budgets, Cost Savings, Heart Transplantation

## Abstract

**Background:**

Polypharmacy is a significant economic burden.

**Objective:**

We tested whether using reverse auction (RA) as compared with commercial pharmacy (CP) to purchase medicine results in lower pharmaceutical costs for heart failure (HF) and heart transplantation (HT) outpatients.

**Methods:**

We compared the costs via RA versus CP in 808 HF and 147 HT patients followed from 2009 through 2011, and evaluated the influence of clinical and demographic variables on cost.

**Results:**

The monthly cost per patient for HF drugs acquired via RA was $10.15 (IQ 3.51-40.22) versus $161.76 (IQ 86.05‑340.15) via CP; for HT, those costs were $393.08 (IQ 124.74-774.76) and $1,207.70 (IQ 604.48-2,499.97), respectively.

**Conclusion:**

RA may reduce the cost of prescription drugs for HF and HT, potentially making HF treatment more accessible. Clinical characteristics can influence the cost and benefits of RA. RA may be a new health policy strategy to reduce costs of prescribed medications for HF and HT patients, reducing the economic burden of treatment.

## Introduction

It is estimated that there are 5.1 million patients with heart failure (HF) in the United States of America (USA)^1^ and 6.4 million patients in Brazil^2^. Heart failure was the single most frequent cause of hospitalization in the elderly population in Brazil^2^. Pharmacological therapy-oriented guidelines reduce progression, morbidity, mortality, and hospitalization in HF^3,4^. Public and private spending on pharmaceuticals account for a substantial fraction of the total expenses of health care in developed and developing countries^5^. Heart failure is a cardiovascular disease with estimated costs of $32.4 billion in the USA in 2015^6^. Approximately 3% of the total cost of HF was expended on medications costing approximately $1.11 billion in 2009^7^. Surprisingly, despite the impact on HF costs, few studies have examined the pharmacological costs in patients with HF and the influence of demographic and clinical characteristics^8-12^. In addition, those few studies do not reflect contemporary practice. No previous study examined the influence of additional procedures, such as heart transplantation (HT).

Health resources are scarce, but the needs are unlimited. Remarkably, strategies for reducing the cost of pharmacological HF treatments have not been tested, despite the impact of drug costs on government budgets and noncompliance with the use of medications. Studies regarding pharmacological treatments and their costs can provide a rationale for government policies to plan financial resources, and are essential tools for pharmacoeconomics in public health. Therefore, the purpose of this study was to compare the costs of the pharmacological treatment of HF and HT via reverse auction (RA) *versus* estimated costs in commercial pharmacies (CP).

## Methods

### Study population

We retrospectively obtained the clinical, demographic, and pharmacological treatment data of all patients diagnosed with HF or of HT recipients consecutively managed at the Heart Failure Outpatient Clinic of Heart Institute of the Hospital das Clínicas da Faculdade de Medicina da Universidade de São Paulo in Brazil. Demographic data collected included ethnicity escribed according to a previous study^13^, age, sex, comorbid condition, cause of HF, left ventricular ejection fraction (LVEF), number of ambulatory visits, medications, and most frequent stage of HF during the study period. The LVEF was obtained by use of any method, such as echocardiography, radionuclide ventriculography, cardiac magnetic resonance, or ventriculography via cardiac catheterization. The first LVEF of each patient in the study period was considered. The patients were being cared for by physicians and by a multidisciplinary team specialized in HF and HT from January 2009 through April 2011. Patients older than 16 years and on ambulatory care were included, except for one 9-year-old HT patient. Patients with incomplete clinical or cost information and any prescription drugs received during hospitalization were excluded.

### Study design

Data relating to outpatient visits are stored in electronic health records so that any electronic medical prescription is automatically generated through an automated data-entry process. Medications are delivered every month to patients according to the last validated medical prescription. We retrieved information about all the medications each patient received, and the investigators reviewed all the medication information to ensure consistency.

At our institution, which is a public body, drugs are purchased via RA. Reverse auction, is a mechanism through which, once consolidated the demand of the participating entities and established the technical characteristics of the products to be purchased, those with the best price are selected through electronic biddings towards the lowest. This guarantees a fair and transparent competition, and the most inexpensive prices of the market, especially in Latin American countries, such as Brazil. Purchases are financed by an annual budget assigned to each public body by public health funds. In procurement auctions for pharmaceuticals, suppliers bid for a very detailed contract of medicine supply, which specifies the drug, quantity, place and time schedule to delivery^14^.

A procurement contract specifies a very detailed drug to be supplied, which is a unique combination of active ingredient, form, concentration, number of units, and packing. Public bodies are forbidden to procure a drug of a specific brand. They are obliged to acquire the drug from the lowest bid's supplier, which, in principle, can be a generic or a branded drug^14^.

The medications are delivered gratuitously to patients treated at a public hospital according to the prescription. We compared the pharmacological treatment of HF and HT purchased via RA *versus* estimated costs in the private market in CP. The price of CP was defined using Brasíndice Pharmaceutical Guide, an official federal index that regulates drug marketing in Brazil (private cost): the Brazilian government sets maximum retail prices practiced by the pharmacies. The cost of each pill was based on the last institutional purchase in 2011 to avoid differences in prices between time periods. Value cost was converted into American dollars according to the market exchange rate of $0.50815 on April 12, 2013, to establish the values in a stable currency. The monthly cost per patient was calculated.

Drugs included were beta-blockers, diuretics, calcium channel blockers (CCB), angiotensin-converting enzyme inhibitors (ACEI), angiotensin receptor blockers (ARB), other cardiovascular drugs (OCD), lipid-lowering drugs, antiplatelet drugs, antiarrhythmic drugs, nitrates, anticoagulants or inhibitors of platelet adenosine diphosphate receptor (ACG-IPADPR), digitalis, immunosuppressants and other noncardiovascular drugs (ONCD). Drugs typically used for short course treatments (antibiotics) or on an as-needed basis (analgesics or sublingual nitroglycerin) were also included.

We further analyzed costs according to clinical and demographic variables. The study protocol was approved by the Ethics Committee of the Instituto do Coração de São Paulo (registration number 22831813.2.0000.00680), which waived the need for patient consent because no identifying participant information was obtained.

### Statistical analysis

Measurement data are reported as mean ± standard deviation (SD) for variables normally distributed, and as medians within the interquartile range (IQ) for variables not normally distributed or as frequencies with percentages for all categorical variables. Univariate analysis was done with the chi-square or Fisher exact test to compare categorical variables. Normally distributed continuous variables were compared using the Student *t* test, and, for those non normally distributed, the Kruskal-Wallis test was used to assess differences between variables. Significant results demonstrated by the Kruskal-Wallis test were further analyzed for significance with the least significant difference (LSD) multiple-comparison *post hoc* test. To determine the correlation between continuous variables, we applied the correlation test according to normality distribution (*r*). The LVEF was analyzed in cohorts stratified into ≤ 40% and > 40%.

The monthly cost per patient was log transformed. Multivariate analysis was performed with Generalized Estimating Equations (GEE), and the results expressed as nonstandardized coefficients (β). Generalized Estimating Equations were applied using the continuous variables (age, LVEF, and ambulatory appointments), and the categorical variable 'cause of HF' as fixed effects. Comorbidities, functional class, ethnicity and sex were used as random effects. The advantage of this approach is that it used all available data and adjusted results based on correlations between outcomes and predictor variables. We used the criterion Wald chi-square test for choosing the best structure to be adopted in the model concerned. The quasi likelihood ratio test was also used to compare adjusted models.

Statistical analysis was performed with SPSS 17.0. p-values < 0.05 were considered significant, and p-values < 0.10, as a trend.

## Results

**Table 1:** Baseline characteristics of the study population

**CHARACTERISTICS**	**HF n = 808 n (%)**	**HT n = 147 n (%)**
**Age, years**	56.5 ± 12.2	49.9 ± 16.6
< 20	5(0.6)	9(6.1)
20 to 40	80 (9.9)	37 (25.2)
41 to 65	427 (52.8)	57 (11.8)
≥ 66	296 (36.6)	44 (29.9)
**Ambulatory appointment**		
< 5	250 (30.9)	16 (10.9)
6 to 10	302 (37.4)	17 (11.6)
11 to 15	127 (15.7)	42 (28.6)
16 to 20	48 (5.9)	28 (19.0)
≥ 21	81 (10.0)	44 (29.9)
**Ethnicity**		
White and yellow	619 (78.3)	127 (86.4)
Black and mulatto	172 (21.7)	20 (13.6)
**Cause**		
Ischemic	141 (30.7)	18 (12.9)
Other causes	232 (50.5)	73 (52.5)
Chagasic	86 (18.7)	48 (34.5)
**Sex**		
Male	508 (62.9)	101 (68.7)
Female	300 (37.1)	46 (31.3)
**Comorbidities**		
Hypertension	157 (19.5)	40 (27.2)
Diabetes	150 (18.6)	30 (20.4)
Cerebrovascular accident	31 (3.9)	5 (3.4)
Renal Failure	56 (7.0)	18 (12.2)
COPD	13 (1.6)	2 (1.4)
Myocardial revascularization	20 (2.5)	0 (0.0)
Coronary angioplasty	9 (1.1)	2 (1.4)
Myocardial infarction	56 (7.0)	4 (2.7)
**Prescribed cardiovascular drug**		
Beta-blocker	703 (87.1)	40 (27.2)
Diuretic	703 (87.1)	54 (36.7)
CCB	205 (25.4)	114 (77.6)
ACEI, ARB	573(71.0)	54 (36.7)
Lipid-lowering	595 (73.7)	117 (79.6)
Antiplatelet drug	354 (43.9)	30 (20.4)
ACG or IPADPR	212 (26.3)	20 (13.6)
Antiarrhythmic drug	85 (10.5)	4 (2.7)
Digitalis	309 (38.3)	15 (10.2)
Nitrate	197 (24.4)	11 (7.5)
OCD	330 (40.9)	47 (32.0)
**Prescribed non-cardiovascular drug**		
Antibiotic	42 (5.2)	68 (46.3)
Immunosuppressant	0 (0)	147 (100)
ONCD	630 (78.1)	147 (100)

HF: heart failure; HT: heart transplantation; COPD: chronic obstructive pulmonary disease; ACG: anticoagulants; IPADPR: inhibitor of platelet adenosine *diphosphate* receptor; ONCD: other noncardiovascular drugs; beta-blockers (atenolol, bisoprolol, metoprolol, carvedilol, propranolol, sotalol); diuretics (furosemide, hydrochlorothiazide, chlorthalidone, spironolactone); CCB: calcium channel blockers (diltiazem, verapamil, amlodipine, nifedipine, losartan); ACEI: angiotensin-converting enzyme inhibitors (captopril, enalapril, lisinopril); ARB: angiotensin receptor blockers, lipid-lowering drugs (atorvastatin, ciprofibrate, ezetimibe, rosuvastatin, simvastatin); antiplatelet drugs (aspirin); ACG or IPADPR (enoxaparin, warfarin, clopidogrel); antiarrhythmic drug (amiodarone, propafenone); digitalis (digoxin); nitrates (isosorbide, propatylnitrate); OCD: other cardiovascular drugs (clonidine, doxazosin, hydralazine, methyldopa).

In the HF group, the mean time of study follow-up was 16.7 ± 8.8 months, with a median number of eight ambulatory appointments for each patient, whereas, in the HT group, the study follow-up was 23.1 ± 5.8 months, and the median number of ambulatory appointments for each patient was 16. A total of 8,448 medical prescriptions were analyzed in HF patients, and 3,217 medical prescriptions in HT recipients. Complete cost data could not be retrieved in 82 (10.1%) patients with HF and in 1 (0.7%) HT recipient, and these patients were excluded. Ambulatory appointments were more frequent among HF patients in functional class III and IV as compared with those in class I and II. In patients with functional class III and IV, 26.8% of the cause was chagasic, 34.1% ischemic, and 39% other causes, compared with 12.9%, 29.6%, and 57.5%, respectively, in patients with functional class I and II (p = 0.003).

### Drugs prescribed for HF

The most frequently prescribed drugs in descending order were beta-blockers, diuretics, ONCD, lipid-lowering drugs, ACEI-ARBs (Table 1). Prescribed drugs differed between patients with LVEF ≤40% and those with LVEF > 40%. Beta-blockers, diuretics, antiplatelet drugs, antiarrhythmic drugs and nitrates were more frequently prescribed for HF patients with LVEF ≤ 40%, whereas CCB prescriptions were more frequent for patients with LVEF > 40% (Table 2). Beta-blockers (75.9%, p = 0.009), diuretics (75.9%, p = 0.009), ACEI-ARB (76.6%, p = 0.034), nitrate (61.9%, p = 0.001) and OCD (68.8%, p = 0.036) were more frequently prescribed in functional classes I and II. Patients of white and yellow ethnicity received more OCD (74.1%, p = 0.015) and antiplatelet drugs (82.2%, p = 0.018). Calcium channel blockers (60.3%, p < 0.001) and lipid-lowering drugs (47%, p < 0.001) were more frequently prescribed for other causes, while antiplatelet drugs (53.3%, p < 0.001) and nitrates (43%, p < 0.001) were more frequently prescribed for ischemic patients, and antiarrhythmic drugs (41%, p < 0.001) for chagasic patients. ACEI-ARB (33.3%, p = 0.001), OCD (42.1%, p = 0.013), antiplatelet drugs (31.4%, p = 0.003) and ONCD (40.2%, p = 0.001) were less frequently prescribed for female patients.

**Table 2:** Profile of prescribed drugs according to left ventricular ejection fraction

**Prescribed drug**	**HF**			**HT**		
	**LVEF ≤ 40% (n = 321) n (%)**	**LVEF > 40% (n = 134) n (%)**	**p**	**LVEF ≤ 40% (n = 10) n(%)**	**LVEF > 40% (n = 126) n (%)**	**p**
Beta-blocker	287 (89.4)	109(81.3)	0.020	5(50.0)	32 (25.4)	0.13
Diuretic	287(89.4)	109(81.3)	0.020	4(40.0)	48(38.1)	1.00
CCB	67(20.9)	43(32.1)	0.011	7(70.0)	102(81.0)	0.41
ACEI, ARB	221(68.8)	90(67.2)	0.72	6(60.0)	46(36.5)	0.18
OCD	153(47.7)	55(41.0)	0.19	5(50.0)	41(32.5)	0.30
Lipid-lowering	241(75.1)	89(27.0)	0.059	7(70.0)	106(84.1)	0.37
Antiplatelet	154(48.0)	44(32.8)	0.003	3(30.0)	24(19.0)	0.41
ACG or IPADPR	93(29.0)	39(29.1)	0.97	1(10.0)	19(15.1)	1.00
Antiarrhythmic	44(13.7)	9(6.7)	0.034	0(0.0)	3(100)	NP
Digitalis	127(39.6)	44(32.8)	0.17	0(0.0)	15(11.9)	NP
Nitrate	104(32.4)	24(17.9)	0.002	3(30.0)	7(5.6)	0.026
Antibiotic	25(7.8)	7(5.2)	0.33	5(50.0)	62(49.2)	1.00

HF: heart failure; HT: heart transplantation; EF: ejection fraction; NP: not possible; ACG: anticoagulants; IPADPR: inhibitors of platelet adenosine diphosphate receptor; beta-blockers (atenolol, bisoprolol, metoprolol, carvedilol, propranolol, sotalol); diuretics (furosemide, hydrochlorothiazide, chlorthalidone, spironolactone); CCB: calcium channel blockers (diltiazem, verapamil, amlodipine, nifedipine, losartan); ACEI: angiotensin-converting enzyme inhibitors (captopril, enalapril, lisinopril); ARB: angiotensin receptor blockers; OCD: other cardiovascular drugs (clonidine, doxazosin, hydralazine, methyldopa); lipid-lowering drugs (atorvastatin, ciprofibrate, ezetimibe, rosuvastatin, simvastatin); antiplatelet drug (aspirin), ACG or IPADPR (enoxaparin, warfarin, clopidogrel); antiarrhythmic drugs (amiodarone, propafenone), digitalis (digoxin); nitrates (isosorbide, propatylnitrate).

In the HF group, men consumed lower doses of atenolol, captopril, losartan, spironolactone, and isosorbide, and higher doses of carvedilol and hydralazine, but equal doses of enalapril, although the difference was not statistically significant among daily prescribed medication doses between sexes. In respect to functional class, the median doses of enalapril in functional class III and IV and also I and II were 30.28 mg/daily (IQ 14.12-35.79) and 33.85 mg/daily (IQ 22.00-38.67), respectively (p = 0.014). The median doses of captopril were 39.58 mg/daily (IQ 13.54-107.29) and 77.52 mg/daily (IQ 43.23-146.42), respectively (p = 0.059). Regarding ethnicity, African-Brazilians (black and mulatto) used more enalapril (35.42 mg/daily, IQ 23.61-39.27) than white and yellow Brazilians (32.93 mg/daily, IQ 18.33-37.78) (p = 0.039). Concerning age, 41 to 65 year-old patients with HF more frequently received enalapril (34.29 mg/daily, IQ 20.21-38.65, p = 0.019) and spironolactone (25.93 mg/daily, IQ 24.24-30.56, p = 0.049), a trend toward higher doses of carvedilol (48.08 mg/daily, IQ 35.04-62.50, p = 0.076) and captopril (93.05 mg/daily, IQ 66.67-145.4, p = 0.087) being observed. Regarding cause, lower doses of carvedilol (42.11 mg/daily, IQ 25.00-50.00, p < 0.001) and enalapril (23.33 mg/daily, IQ 14.44-32.22, p < 0.001) were administered to chagasic patients as compared to ischemic patients and those with heart disease of other causes, and lower doses of losartan (85.55mg/daily, IQ 52.77-100.00, p = 0.021) were administered to chagasic and ischemic patients as compared to those with heart disease of other causes. The correlations between daily consumption of carvedilol and LVEF were weak (r =.125, p = 0.017).

**Figure 1:**
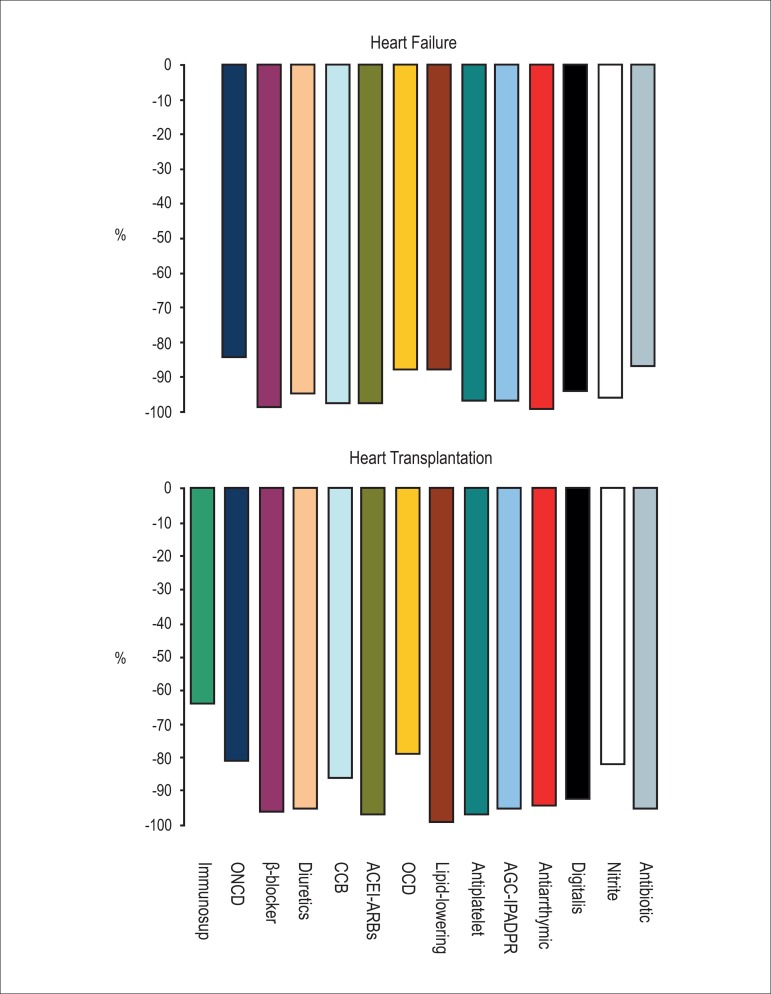
Percentage cost reduction in delivered prescription drugs purchased via reverse auction in comparison with private costs according to each drug group in heart failure (Top) and heart transplantation (Bottom). Immunosup: immunosuppressants; ONCD: other noncardiovascular drugs; CCB: calcium channel blockers; ACEI: angiotensin-converting enzyme inhibitors; ARB: angiotensin receptor blockers; OCD: other cardiovascular drugs; Antiplatelet: antiplatelet drugs; ACG: anticoagulants; IPADPR: inhibitors of platelet adenosine diphosphate receptor.

In the HT group, immunosuppressants, ONCD, lipid-lowering drugs and CCB were most frequently prescribed (Table 1). Additionally, there was a difference regarding the use of nitrates between patients with LVEF ≤ 40% and those with LVEF > 40% (p = 0.026) (Table 2). Patients of white and yellow ethnicity received more antibiotics (77.9%, p = 0.006). With respect to cause, differences were not statistically significant among the drugs used. Concerning sex, antibiotics were more frequently used by men than by women (58.8%, p = 0.016). Figure 1 shows the magnitude of cost reduction for drugs purchased via RA.

### Cost of pharmacological treatment via reverse auction

In HF patients, the total cost was $534,010.20 (n = 726), and the median monthly cost per patient was $10.15 (IQ 3.51-40.22). The most costly classes of drugs in decreasing order of value were ONCD, lipid-lowering drugs and OCD (Table 3).

**Table 3:** Total cost in dollars of prescribed drugs for heart failure and heart transplantation study populations

**Prescribed drug**	**HF**				**HT**			
	**Reverse Auction**	**Reverse Auction**	**Private**	**Private**	**Reverse Auction**	**Reverse Auction**	**Private**	**Private**
	**Total**	**%**	**Total**	**%**	**Total**	**%**	**Total**	**%**
Beta-blocker	5,587.37	1.05	594,268.42	14.89	600.39	0.03	13,832.19	0.24
Diuretic	7,656.28	1.43	146,626.03	3.67	368.85	0.02	8,441.09	0.15
CCB	1,212.11	0.23	58,448.57	1.46	6,847.05	0.38	51,373.17	0.90
ACEI, ARB	16,905.47	3.16	209,315.46	5.24	2,102.12	0.12	15,675.55	0.27
OCD	24,174.38	4.52	162,075.63	4.06	4,846.76	0.27	15,933.67	0.28
Lipid-lowering	79,108.02	14.80	196,806.89	4.93	6,267.88	0.35	66,905.15	1.17
Antiplatelet	566.45	0.11	19,825.63	0.50	67.07	0.00	2,347.49	0.04
ACG or IPADPR	7,911.35	0.15	58.458,19	1.46	662.50	0.04	9,667.25	0.17
Antiarrhythmic	1,993.28	0.37	17,018.91	0.43	40.55	0.00	729.86	0.01
Digitalis	672.34	0.13	15,980.01	0.40	18,70	0.00	336.58	0.01
Nitrate	5,300.55	0.99	37,563.87	0.94	559.03	0.03	1,799.95	0.03
Antibiotic	217.40	0.04	5,880.07	0.15	6,647.44	0.37	199,964.37	3.49
Immunosuppressant	-	-	-	-	1,540,724.53	86.20	4,159,017.41	72.63
ONCD	390,244.35	73.02	2,468,908.68	61.86	217,709.30	12.18	1,179,687.44	20.60
TOTAL	534,428.49	100	3,991,176.38	100	1,787,462.17	100	5,725,965.20	100

HF: heart failure; HT: heart transplantation; ACG: anticoagulants; IPADPR: inhibitors of platelet adenosine diphosphate receptor; ONCD: other noncardiovascular drugs; beta-blockers (atenolol, bisoprolol, metoprolol, carvedilol, propranolol, sotalol); diuretics (furosemide, hydrochlorothiazide, chlorthalidone, spironolactone); CCB: calcium channel blockers (diltiazem, verapamil, amlodipine, nifedipine, losartan); ACEI: angiotensin-converting enzyme inhibitors (captopril, enalapril, lisinopril); ARB: angiotensin receptor blockers; lipid-lowering drugs (atorvastatin, ciprofibrate, ezetimibe, rosuvastatin, simvastatin); antiplatelet drug (aspirin), ACG or IPADPR (enoxaparin, warfarin, clopidogrel); antiarrhythmic drugs (amiodarone, propafenone); digitalis (digoxin); nitrates (isosorbide, propatylnitrate); OCD: other cardiovascular drugs (clonidine, doxazosin, hydralazine, methyldopa).

On the other hand, the total cost for HT recipients was $1,787,462.17 (n = 146), and the median monthly cost per patient was $393.08 (IQ 124.74-774.76). The most costly classes of drugs were immunosuppressants and ONCD (Table 3).

### Cost of pharmacological treatment via private market

In HF patients, the estimated total cost was $3,991,176.38 (n = 726), and the median monthly cost per patient was $161.76 (IQ 86.05-340.15). The most costly classes of drugs in decreasing value were ONCD and beta-blockers (Table 3).

For HT patients, the total cost was $5,725,965.20 (n = 146), and the median monthly cost per patient was $1,207.70 (IQ 604.48-2,499.97). Not surprisingly, the most costly classes of drugs were immunosuppressants and ONCD (Table 3).

### Cost via reverse auction according to subgroup analyses

**Figure 2:**
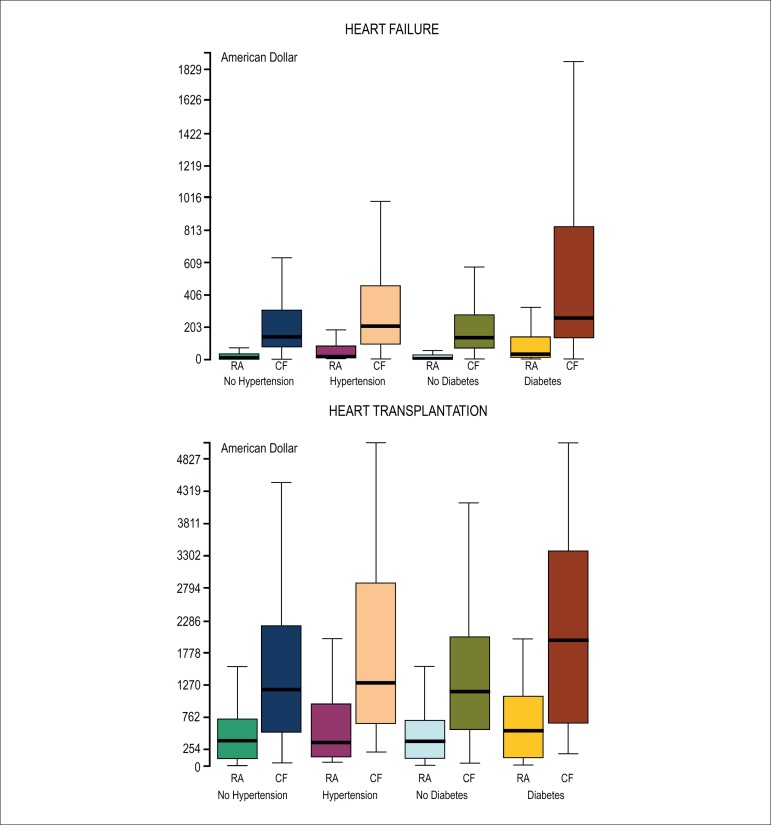
Cost of delivered prescription drugs purchased via reverse auction (RA) compared with commercial pharmacy (CP) costs according to the diagnosis of systemic arterial hypertension and diabetes mellitus in heart failure and heart transplantation.

The monthly median cost per HF patient was $9.74 (IQ 3.48-33.65, n = 453) for men and $12.24 (IQ 3.57-44.01, n = 273) for women (p = 0.127). An increment in monthly median cost for HF was observed in hypertension (p < 0.001) and diabetes (p < 0.001). Hypertension was responsible for an incremental cost of $8.02 (48% higher) (p < 0.001) and diabetes, for an incremental cost of $24.35 (76% higher) (p < 0.001). The cost for ischemic cardiomyopathy ($16.84, IQ 6.32-16.84) was significantly higher than that for Chagas' disease ($8.53, IQ 2.98-26.52, p < 0.001) and other causes ($6.79, IQ 3.21-18.58, p < 0.001). The lower cost for Chagas' disease may be due to the fact that chagasic patients may not tolerated full doses and doctors are not encouraged to use full doses because there is no trial in Chagas' disease. In the multivariate analysis, older HF subjects had significantly higher costs (β = .021, p < 0.001), and patients without hypertension and diabetes had significantly lower costs (β = -.517, p < 0.001; β = -.979, p < 0.001). The magnitude of cost reduction via RA was higher in diabetes and hypertension (Figure 2).

In the HT group, the monthly median cost per patient was $365.17 (IQ 100.83-683.59, n = 101) for men and $435.58 (IQ 248.40-920.86, n = 45) for women (p = 0.268). None of the comorbidities caused an incremental cost. Differences were statistically significant between the costs of patients with a previous history of chagasic cardiomyopathy ($271.71, IQ 66.49-568.54) and other causes ($472.40, IQ 182.05-888.53, p = 0.009). Additional parallels between clinical variables and monthly median cost for both groups are shown in Table 4. In multivariate analysis, HT patients without hypertension had lower cost (β = -.324, p < 0.001). In this model, other variables were not statistically significant in predicting cost. The magnitude of cost reduction via RA was higher in diabetes and hypertension (Figure 2).

**Table 4:** Monthly cost via reverse auction or commercial pharmacies of prescribed drugs for heart failure and heart transplantation study populations according to clinical and demographic variables

**CHARACTERISTICS**	**HF**				**HT**			
	**n**	**MEDIAN COST**	**IQ 25%-75%**	**p**	**n**	**MEDIAN COST**	**IQ 25%-75%**	**p**
**Reverse auction cost in $**								
**Age, y**				< 0.001				0.19
≤ 19	5	3.16	2.87-24.39		9	732.48	493.62-1,026.12	
20 to 40	68	4.35	2.07-15.63		37	410.07	239.07-844.68	
41 to 65	380	9.8	3.20-45.88		57	365.19	97.16-619.52	
≥ 65	273	13.46	4.80-52.75		43	245.93	90.60-1,021.26	
**Causes**				< 0.001				0.036
Ischemic	132	16.84	6.32-16.84		18	402.49	77.07-932.54	
Chagasic	76	8.53	2.98-26.52		48	271.71	66.49-568.54	
Others	217	6.79	3.21-18.58		73	472.40	182.05-888.53	
**Ambulatory appointment, times**				0.005				0.016
< 5	192	10.84	2.88-39,19		16	576.34	292.84-1,396.98	
6 to 10	286	8.09	3.07-30.27		16	512.22	149.78-1,062.56	
11 to 15	124	15.13	5.23-70.22		42	227.05	73.86-607.01	
16 to 20	46	6.55	2.91-54.35		28	273.05	61.67-654.69	
≥ 21	78	12.89	5.06-41.86		44	493.46	223.14-847.01	
**Functional class**				< 0.001				
I and II	326	7.04	2.82-32.86					
III and IV	101	16.47	5.43-67.55					
**Ethnicity**				0.96				0.34
White and yellow	564	10.07	3.59-10.07		126	390.47	118.43-737.32	
Black and mulatto	148	10.92	3.52-40.47		20	40.47	225.65-1,074.95	
**LVEF**				0.43				0.97
≤ 40%	296	11.82	3.47-47.80		10	358.93	86.11-957.32	
> 40%	119	9.46	3.72-27.61		126	390.47	124.75-740.92	
**Private cost in $**								
**Age, y**				< 0.001				0.52
≤ 19	5	105.45	52.69-520.48		09	1,851.58	1,284.76-2,755.11	
20 to 40	68	114.52	62.57-188.12		37	1,234.25	839.74-2,455.04	
41 to 65	380	148.90	79.68-331.97		57	1,155.17	556.26-2,491.22	
≥ 65	273	193.19	113.57-420.72		43	1,004.99	572.62-2,734.57	
**Causes**				0.004				0.14
Ischemic	132	200.96	113.73-447.87		18	1,581.77	554.10-3,141.53	
Chagasic	76	138.85	19.03-303.53		48	1,024.00	355.21-1,553.49	
Others	217	139.82	84.39-274.33		73	1,378.35	784.73-2,485.21	
**Ambulatory appointment, times**				0.002				0.009
< 5	192	168.48	67.85-321.16		16	1,507.97	984.70-3,821.39	
6 to 10	286	147.90	79.49-308.74		16	1,530.34	713.99-2,890.81	
11 to 15	124	190.69	111.44-510.07		42	800.50	446.29-2,232.58	
16 to 20	46	151.78	89.09-339.14		28	905.63	309.77-1,756.99	
≥ 21	78	189.04	116.25-363.56		44	1,445.44	955.98-2,737.73	
**Functional class**				0.010				
I and II	326	134.27	72.15-278.23					
III and IV	101	213.35	110.97-637.56					
**Ethnicity**				0.57				0.15
White and yellow	564	169.05	85.18-339.40		126	1,161.89	573.85-2,432.95	
Black and mulatto	148	157.10	86.02-391.50		20	1,709.93	800.58-3,134.78	
**LVEF**				0.92				0.32
≤ 40%	310	149.32	75.75-331.42		10	1,133.99	712.47-2,548.44	
> 40%	129	136.30	72,65-286.64		126	1,203.64	583.40-2,487.27	

Measurement data are presented as median with interquartile range. LVEF: left ventricular ejection fraction; HF: heart failure; HT: heart transplantation.

### Cost via commercial pharmacies according to subgroup analyses

In the HF group, the monthly median cost for men was $155.34 (IQ 91.30-314.85) and for women $175.21 (IQ 80.68-415.44) (p = 0.412). With respect to comorbidities, an incremental monthly median cost was associated with hypertension (p < 0.001) and diabetes (p < 0.001). Hypertension was responsible for an incremental cost of $60.35 (28% higher) (p = 0.005), and diabetes, for an incremental cost of $130.19 (48% higher) (p < 0.001). The cost of ischemic cardiomyopathy ($200.96, IQ 113.73-447.87) was significantly higher than that of chagasic cardiomyopathy ($138.85, IQ 19.03-303.53, p = 0.003) and other causes ($139.82, IQ 84.39-274.33, p = 0.004). In the multivariate analysis, a significant interactive effect between cost and age was observed for HF patients; not surprisingly, older subjects had significantly higher costs (β = .018, p < 0.001). Men (β = -.165, p < 0.001) and subjects without diabetes had significantly lower costs (β = .568, p = 0.003).

In the HT group, a tendency toward a lower monthly median cost for prescribed drugs was observed for men ($1,056.83, IQ 532.31-2,325.00) than for women ($1,313.48, IQ 907.33-3,011.20) (p = 0.069). Nevertheless, diabetes was the only comorbidity responsible for an incremental cost of $795.99 (40% higher) (p = 0.045). No difference in cost was observed between causes. Additional parallels between clinical variables and monthly median cost for both groups are shown in Table 4. In the HT group, the multivariate analysis showed a significant interactive effect for cost and absence of hypertension (β = -.218, p < 0.001). The absence of hypertension resulted in lower costs in the private system. In this model, there were no statistically significant differences for other variables.

## Discussion

To our knowledge, this is the first study to demonstrate that the cost of delivered prescription drugs in HF and HT, patients purchased via RA is remarkably lower than estimated private market costs in the real world scenario of clinical practice. In fact, in this comparison, purchasing via RA for HF followed by delivery to patients is likely a bargain. Older age was associated with higher costs, and the absence of diabetes was associated with lower costs in HF patients via both purchasing systems. The magnitude of cost reduction via RA was higher in diabetes and hypertension (Figure 2).

Our cost for HF pharmacological treatment was expressively lower in comparison with the previously reported cost, ranging from $261 to $438 per month per patient^9,10^. Actually, the cost of drugs purchased via RA in our investigation ranged from approximately 2.28% to 3.8% of previously published data from the USA, whereas the estimated cost in the private market ranged from 37% to 62%. The USA health system, acknowledged as one of the most expensive in the world with consequently higher prices for medications^15^, could contribute for those differences. However, the enormous difference in cost corroborated our findings of the RA effectiveness in reducing costs for HF treatment.

Regarding HT, the comparison with data of only one published study analyzing the cost of immunosuppressants from solid organ transplantation up to 2 years post-transplantation shows that RA may be proportionally less effective in reducing costs after HT. In that study, costs ranged from $448 to $1,321 per patient per month^16^. However, the cost of other medications was not considered, which could underestimate the total cost of pharmacological treatment.

To explain our results, we would like to speculate that purchasing via RA might create a competitive environment for many industries involved in producing HF medications. The competition, rather than being a disadvantage to industry, would push pharmaceutical manufacturers to improve their business processes to maintain a profit while at the same time benefiting society. Conversely, the small number of industries involved in the production of immunosuppressant drugs and the restricted market may prevent the development of a competitive environment and limit the effectiveness of RA for HT. As a result, the pharmacological therapy for transplantation has a high cost as compared with that for HF^16^.

As expected and confirming published data, comorbidities were associated with a higher cost^8,9^. The higher cost for older patients could be explained by the ageing process leading to greater comorbidity. In general, comorbidities require polypharmacy treatment; however, it is worth noting that diabetes was associated with higher incremental costs in HF, probably because of the role of diabetes in the development of many comorbidities^9^. However, RA was more effective in diabetes (Figure 2).

### Clinical and health systems implications

Our findings suggest that RA is a positive alternative for health system financial support and could be introduced to other countries to reduce the cost of the pharmacological treatment of HF and after HT. People involved in medication supply for HF should reflect about the possibility of purchase via RA and delivery to patients. The cost of outpatient medications may influence the patients' adherence to recommended pharmacological treatment^10^. The lower cost could result in greater access to pharmacological treatment with greater adherence to prescribed medications, better survival and less hospitalization. The worldwide use of RA to health policies could benefit many people in developed and mainly undeveloped countries, reducing the economic burden of HF.

### Limitations

Our study has limitations. We assessed only prescribed and delivered drugs; consequently, we were unable to ascertain patient adherence. However, it is sufficient to demonstrate how much pharmacological therapy occurs in clinical practice. The regimens administered to the patients were based on those prescribed by specialized cardiologists at a tertiary center and may not represent primary care in HF treatment. Our single-center study did not include patients from multiple practices and diverse Brazilian geographic regions; however, in these conditions, the pharmacological treatment is not usually guideline-oriented. Finally, our study was a retrospective review of medical records, because a prospective study would require more time and economic resources.

## Conclusion

In the present study in contemporary practice, we were able to demonstrate that RA may be valuable as a potential tool for reducing HF burden dependent on the pharmacological therapy cost of HF outpatients and after HT. In addition, the prevalence of comorbidities and older age are associated with higher cost, which should be considered in planning health strategies for HF. Likewise, the key finding is that RA could become a government strategy to extend drug therapy access to less socially privileged people and in the optimal use of public resources.
